# Factors Influencing Attitudes Toward Dementia Among Elderly Individuals in the Community

**DOI:** 10.1002/alz70858_098125

**Published:** 2025-12-24

**Authors:** Ka Hee Yoo, Su Jeong Seong, Jae Yeon Hwang

**Affiliations:** ^1^ Hallym University Kangdong Sacred Heart Hospital, Seoul, Korea, Republic of (South)

## Abstract

**Background:**

Improving community members' attitudes toward dementia is crucial for creating a dementia‐friendly society where individuals with dementia can live comfortably. Despite its importance, there is limited research on the factors influencing dementia‐related attitudes in community‐dwelling older adults. This study aimed to identify factors influencing attitudes toward dementia among elderly individuals living in the community.

**Method:**

A survey was conducted among randomly selected elderly individuals residing in the community to assess sociodemographic factors, dementia awareness, and attitudes. The Dementia Attitudes Scale (DAS), which consists of the comfort and knowledge subscales, was used to evaluate attitudes. Awareness was assessed through 15 dementia‐related questions designed to evaluate knowledge in a yes‐or‐no format, and depressive symptoms were measured using the Korean version of the Short Geriatric Depression Scale.

**Result:**

Among 247 participants, the comfort subscale of DAS was correlated with depression scores, with more depressive symptoms linked to more negative attitudes. The knowledge subscale of DAS was significantly associated with gender and age, with males and older individuals scoring lower, reflecting more negative attitudes. Participants who recognized that “Drinking alcohol increases dementia risk.” and “Early treatment can slow dementia progression.” showed more positive attitudes on the knowledge subscale. However, those who correctly identified “Dementia is a brain disease.” exhibited more negative attitudes on the comfort subscale.

**Conclusion:**

While knowledge of alcohol as a risk factor and the potential to slow dementia progression through treatment was associated with more positive attitudes, awareness that dementia is a brain disease was linked to lower comfort levels. To improve attitudes toward dementia, it may be more effective to focus on delivering knowledge that positively shapes attitudes rather than providing information indiscriminately.

